# Nucleoside supplementation modulates mitochondrial DNA copy number in the *dguok ^−/−^* zebrafish

**DOI:** 10.1093/hmg/ddy389

**Published:** 2018-11-14

**Authors:** Benjamin Munro, Rita Horvath, Juliane S Müller

**Affiliations:** 1 Wellcome Trust Centre for Mitochondrial Research, Institute of Genetic Medicine, Newcastle University, Newcastle upon Tyne, NE1 3BZ, UK; 2 Department of Clinical Neurosciences, University of Cambridge, John Van Geest Centre for Brain Repair, The ED Adrian Building, Forvie Site, Robinson Way, Cambridge, CB2 0PY, UK

## Abstract

Deoxyguanosine kinase (dGK) is an essential rate-limiting component of the mitochondrial purine nucleotide salvage pathway, encoded by the nuclear gene encoding deoxyguanosine kinase (*DGUOK*). Mutations in *DGUOK* lead to mitochondrial DNA (mtDNA) depletion typically in the liver and brain, causing a hepatocerebral phenotype. Previous work has shown that in cultured *DGUOK* patient cells it is possible to rescue mtDNA depletion by increasing substrate amounts for dGK. In this study we developed a mutant *dguok* zebrafish *(Danio rerio*) line using CRISPR/Cas9 mediated mutagenesis; *dguok^−/−^* fish have significantly reduced mtDNA levels compared with wild-type (wt) fish. When supplemented with only one purine nucleoside (dGuo), mtDNA copy number in both mutant and wt juvenile animals was significantly reduced, contrasting with previous cell culture studies, possibly because of nucleotide pool imbalance. However, in adult *dguok*^−/−^ fish we detected a significant increase in liver mtDNA copy number when supplemented with both purine nucleosides. This study further supports the idea that nucleoside supplementation has a potential therapeutic benefit in mtDNA depletion syndromes by substrate enhancement of the purine nucleoside salvage pathway and might improve the liver pathology in patients.

## Introduction

Mitochondrial DNA depletion syndromes (MDS) are a heterogeneous group of autosomal recessive disorders characterized by a severe reduction in mitochondrial DNA (mtDNA) copy number in different tissues without mutations in the mtDNA itself (1). The initial clinical presentation tends to be organ specific, manifesting in tissues that have a high energy demand such as skeletal muscle, the central nervous system (CNS) and liver, often progressing to severe multisystem disorders (2–4). Reduction of mtDNA leads to the inability to maintain the essential respiratory chain (RC) complexes, resulting in impairment in ATP synthesis, and tissues are unable to function normally (5–7). Changes causing these clinically very heterogeneous diseases are autosomal recessive mutations in at least 15 nuclear genes involved in nuclear-mitochondrial intergenomic signalling pathways. The phenotypes for these disorders can be quite varied from isolated ophthalmoplegia to other tissue-specific or multi-system diseases. The human mitochondrial genome is replicated by polymerase gamma (*POLG*) in concert with other components of the mitochondrial replication machinery (*TWNK, SLC25A4, MGME1, TFAM, DNA2, RNASEH1*) (1). MtDNA depletion in these conditions may be associated with multiple mtDNA deletions and point mutations (*POLG, TWNK, SLC25A4, MGME1, TFAM*). Some genes affecting mtDNA replication may result only in mtDNA deletions or point mutations in adults (*POLG2, DNA2, RNASEH1*), and the inheritance pattern of these genes may be autosomal dominant or recessive (1).

The balanced supply of nucleotides is critically important for DNA integrity. Nucleotide levels are maintained by *de novo* synthesis or by salvage pathways (8). Mutations in nuclear genes involved in the maintenance and supply of mitochondrial nucleotide pools have been associated with MDS with different tissue-specific clinical manifestations (*TK2, DGUOK, RRM2B, TYMP, SUCLA2, SUCLG1*). Early-onset organ-specific autosomal recessive syndromes due to mutations in genes with miscellaneous pathomechanism (*FBXL4, GFER, ABAT, AGK, SLC25A21*) may also result in mtDNA depletion. Furthermore, defects of mtDNA dynamics (*OPA1, MFN2, DRP1*) and quality control (*SPG7, AFG3L2*) can also affect the amount and quality of mtDNA, however, these diseases are more common causes of mtDNA deletions than depletion of mtDNA (1).

Two genes of particular interest are *DGUOK* and *TK2*. *DGUOK* encodes deoxyguanosine kinase (dGK), an important anabolic enzyme involved in the maintenance of mitochondrial purine deoxyribonucleotide triphosphate (dNTP) pools. dGK is the first rate-limiting step of the mitochondrial purine nucleotide salvage pathway, phosphorylating deoxyadenosine (dAdo) and deoxyguanosine (dGuo) into deoxyadenosine monophosphate (dAMP) and deoxyguanosine monophosphate (dGMP), respectively (4). In addition, *TK2*, which encodes thymidine kinase 2 (TK2), has a similar role in maintaining the dNTP pools. TK2 is the first step of the mitochondrial pyrimidine salvage pathway, acting in much the same way as dGK, the difference being that it phosphorylates deoxycytidine (dC) and deoxythymidine (dT) into their corresponding dNMPs, deoxycytidine monophosphate (dCMP) and deoxythymidine monophosphate (dTMP) (9). A noteworthy difference is that mutations in *DGUOK* and *TK2* present clinically as two distinct phenotypes (10). *DGUOK* mutations typically have a hepatocerebral presentation with mtDNA depletion; however, recently chronic progressive external ophthalmoparesis, mitochondrial myopathy, Parkinsonism and multiple mtDNA deletions were also reported in association with recessive *DGUOK* mutations in some patients (11), while mutations in *TK2* have a primarily myopathic manifestation. The involvement of the nervous system often complicates the clinical course of the infantile-onset form while extraocular muscle and facial involvement are characteristics of the late-onset form (12).

The nucleotide salvage pathway recycles free bases from old RNA and DNA in both the mitochondria and cytosol into dNTPs to maintain the nucleotide pools needed for DNA replication. This contrasts with the *de novo* nucleotide synthesis pathway, which occurs only in the cytosol of replicating cells (13). As mtDNA replication is independent of the cell cycle, the salvage pathway is essential in maintaining the mitochondrial dNTP pool in post-mitotic tissue as well as in dividing cells (14–16).

Previously it has been shown that it is possible to rescue mtDNA depletion in *DGUOK* patient myotubes with supplementation of dAMP and dGMP (17). dGK-deficient cell lines (p1, p2) showed a highly significant (*P* < 0.01) increase in mtDNA copy number, reaching normal levels when supplemented with 200 μm dAMP/dGMP or with 400 μm dAMP/dGMP.

A more recent study has shown that mtDNA depletion is rescued by addition of 50 μm dGuo alone to quiescent fibroblast culture and that dAdo addition does not have any detectable effect (18). Supplementation with dGMP also rescued mtDNA depletion. These results indicate that dNMPs are rapidly dephosphorylated in cell culture medium and strongly suggest that dephosphorylation mainly occurs extracellularly. They also illustrate that dGuo is as effective as dGMP for preventing mtDNA depletion in dGK-deficient cells.

Further to this it has been shown that supplementation of deoxythymidine and deoxycytidine monophosphates (dTMP + dCMP) in a murine MDS model with a Tk2 H126N (Tk2^−/−^) knock-in mutation, also showed tissue-specific increases in mtDNA copy number, disease onset delay as well as significantly prolonging the animals’ life span, suggesting bypassing the defective enzyme is an effective therapeutic approach (19). Subsequent work with the same model has shown that supplementation with deoxynucleosides, dC and dT also has the same effect, suggesting that substrate enhancement may be enough to delay the progression of MDS in patients (9).

However, no mouse model has been produced for *DGUOK* mutations yet. Zebrafish (*Danio rerio*) are a good candidate for a disease model as they are routinely used in drug discovery and validation for a variety of neurological and neuromuscular diseases (20,21). Their rapid development, transparency during early life stages and the possibility of administering chemical compounds to tank water make them suitable. The growing use of Crispr/Cas9 technology in zebrafish has made the generation of mutants rapid and reliable and as no *DGUOK* knockout mouse was currently available for supplementation studies, we decided to generate a zebrafish *dguok* mutant strain, and use it to test the substrate enhancement therapy.

## Results

### Generating a mutant zebrafish line for *dguok*

One paralog of the human *DGUOK* gene was located in the zebrafish genome on chromosome 5. The zebrafish *dguok* gene has 7 exons and encodes a protein of 269 amino acids. The amino acid sequence of the zebrafish DGK protein is 55% identical and 72% similar to its human counterpart (Supplementary Material, Fig. S1; accession number NP_001093561.1).

To generate a zebrafish mutant line for *dguok* by using CRISPR/Cas9, we selected two sgRNA target sequences in exon 3 (Supplementary Material, Fig. S1A). We aimed to introduce the mutation as close to the N-terminus of the protein as possible in order to avoid residual enzyme activity to still be present in the mutants. We avoided targeting exon 1, as an alternative translation start might be chosen by the cell, and in exon 2 no sgRNA sequences with high activity and low off-target score could be found. Both selected sgRNA targeting exon 3 produced a range of in frame and frameshift indel mutations. The mutation we chose for our stable mutant line was c.351_352delGCinsCCTG (Supplementary Material, Fig. S1B). The mutation causes a frameshift and induces a premature stop codon after 28 missense amino acids (Supplementary Material, Fig. S1E). In order to verify whether the mutated *dguok* mRNA is degraded by nonsense mediated decay, we analysed *dguok* transcripts from one homozygous mutant adult fish. We were able to amplify and sequence full-length *dguok* transcripts from the liver of the mutant and a wild-type (wt) control. The transcript amplified from the mutant carried the mutation c.351_352delGCinsCCTG homozygously as on genomic DNA (gDNA) level (Supplementary Material, Fig. S1D); no additional modifications such as skipping of exonic sequences or insertion of intronic sequences were detected. Because of the lack of a suitable antibody we were not able to check for the presence of a protein product by western blot. The C-terminal half of the enzyme is well conserved among species and contains a part of the active domain of the enzyme (structure in 22). Therefore we expect that even if the mutated *dguok* mRNA is translated into a protein, this protein would not have the enzymatic activity of dGK.

### Characterization of the mutant phenotype

**Figure 1:**
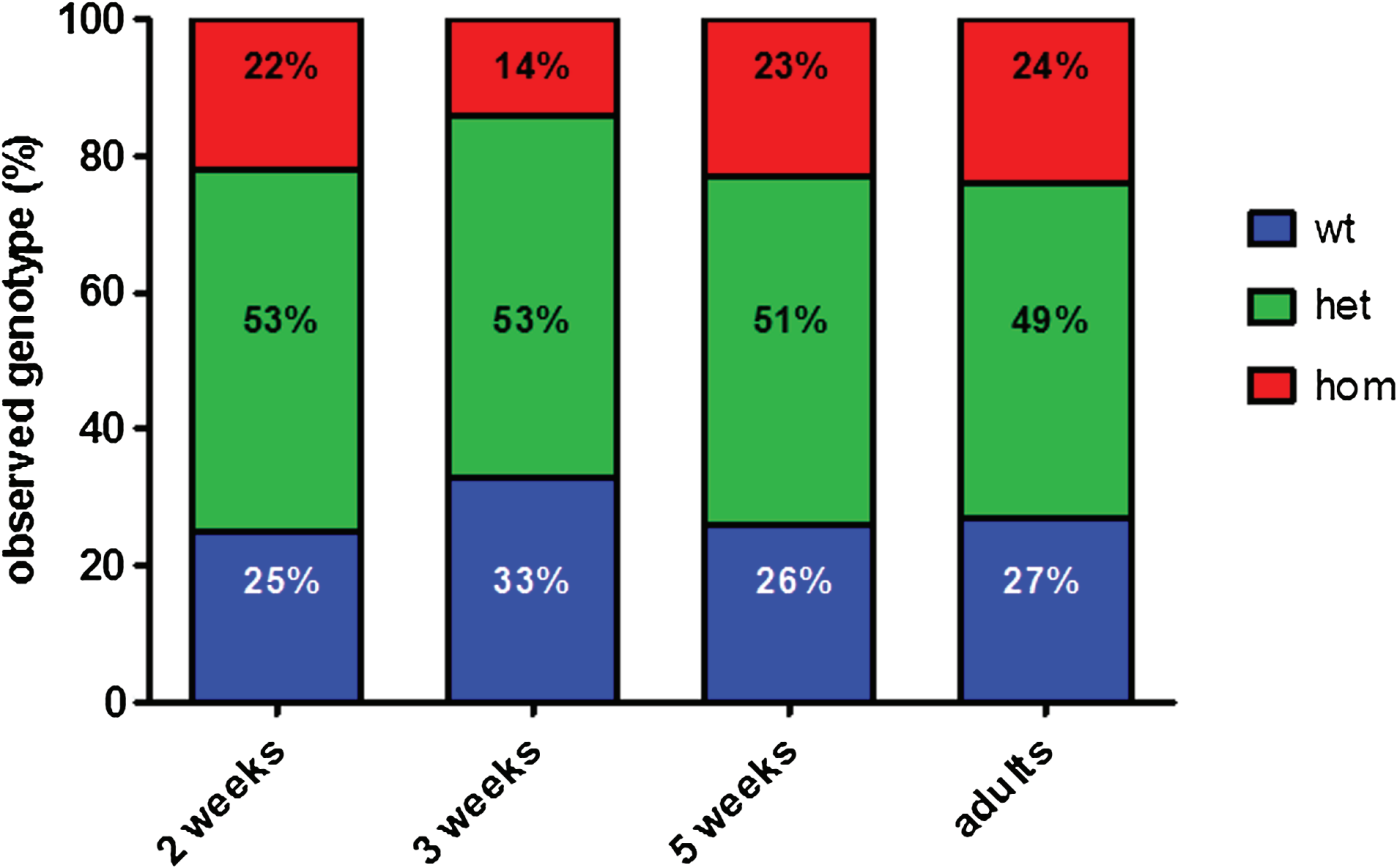
Survival of *dguok* mutant zebrafish. *dguok^−/−^* zebrafish do not exhibit decreased survival initially; the proportion of homozygous mutants among adult population up to the age of 9 months is still close to the expected Mendelian ratio of 25%. The proportion of homozygous and heterozygous fish was determined at the ages of 2, 3, 5 wpf and at the age of 6–9 months. Total numbers of genotyped fish were 159 at 2 wpf, 117 at 3 wpf, 57 at 5 wpf and 146 adult fish.

Surprisingly, embryos and larvae homozygous for *dguok* c.351_352delGCinsCCTG did not display any abnormal phenotype; we did not observe any abnormal morphological traits or swimming defects. When genotyping all offspring of different lays, the mutation was detected at the expected Mendelian rate of 25%, indicating that homozygous mutant animals do not die prematurely during early stages of development (Fig. 1). We attempted to see if blocking the uptake of dNTPs from the cytosol into mitochondria by inhibiting the equilibrative nucleoside transporter 1 (ENT1) with various doses of S-(4-Nitrobenzyl)-6-thioinosine can trigger the appearance of morphological abnormalities in the first 5 days of life. However, we did not observe any morphological abnormalities in either wt, heterozygous or homozygous fish larvae (data not shown).

There was a high variability in terms of size and weight among the mutant animals starting from the age of 3 weeks onwards, but we observed the same variability also among wt controls and heterozygous animals; there was no difference among the groups (Supplementary Material, Fig. S2A). A smaller size was not linked to the homozygous mutant genotype.

In fact, homozygous animals survived into adulthood, and the proportion of homozygous mutants among adult F2 offspring was still ~25% until the age of 9 months (Fig. 1). Adult homozygous fish are indistinguishable from their wt siblings in terms of appearance and size (Supplementary Material, Fig. S2B); they swim normally in the tanks. However, we observed reduced fertility among homozygous fish; we were not able to obtain offspring from a cross of two homozygous adults. On dissection we detected reduced number of eggs in the female fish. At the age of ~10 months, homozygous adults started to struggle to swim normally and maintain their position in the water. This led to them being culled for animal welfare reasons. By 1 year of age all the homozygous adults had died.

### Gene expression analysis in homozygous mutants

We analysed transcript levels of *dguok*, *tk2* and other transcripts encoding mitochondrial proteins in adult wt and homozygous mutant fish in brain, muscle and liver (Supplementary Material, Fig. S3). We designed two primer pairs to amplify *dguok*, one pair located 5′ and the second pair located 3′ of the mutation in order to investigate nonsense mediated decay of the mutated transcript quantitatively. We noticed variability between individual fish also among the tested wt animals, especially in the liver; furthermore, transcript levels also varied between tissues. One of the two wt animals had much higher *dguok* mRNA levels than the other wt animal and the two mutants. Transcripts of *dguok* were detected in all three tissues in the mutant fish, thus excluding relevant nonsense mediated decay of the mutant mRNA (Supplementary Material, Fig. S3). In brain, levels of most transcripts were slightly downregulated in mutants, whereas in tail muscle the same transcripts were slightly increased in mutants. No general trend of up- or down-regulation of the analysed mitochondrial transcripts was observed.

### mtDNA content analysis

**Figure 2:**
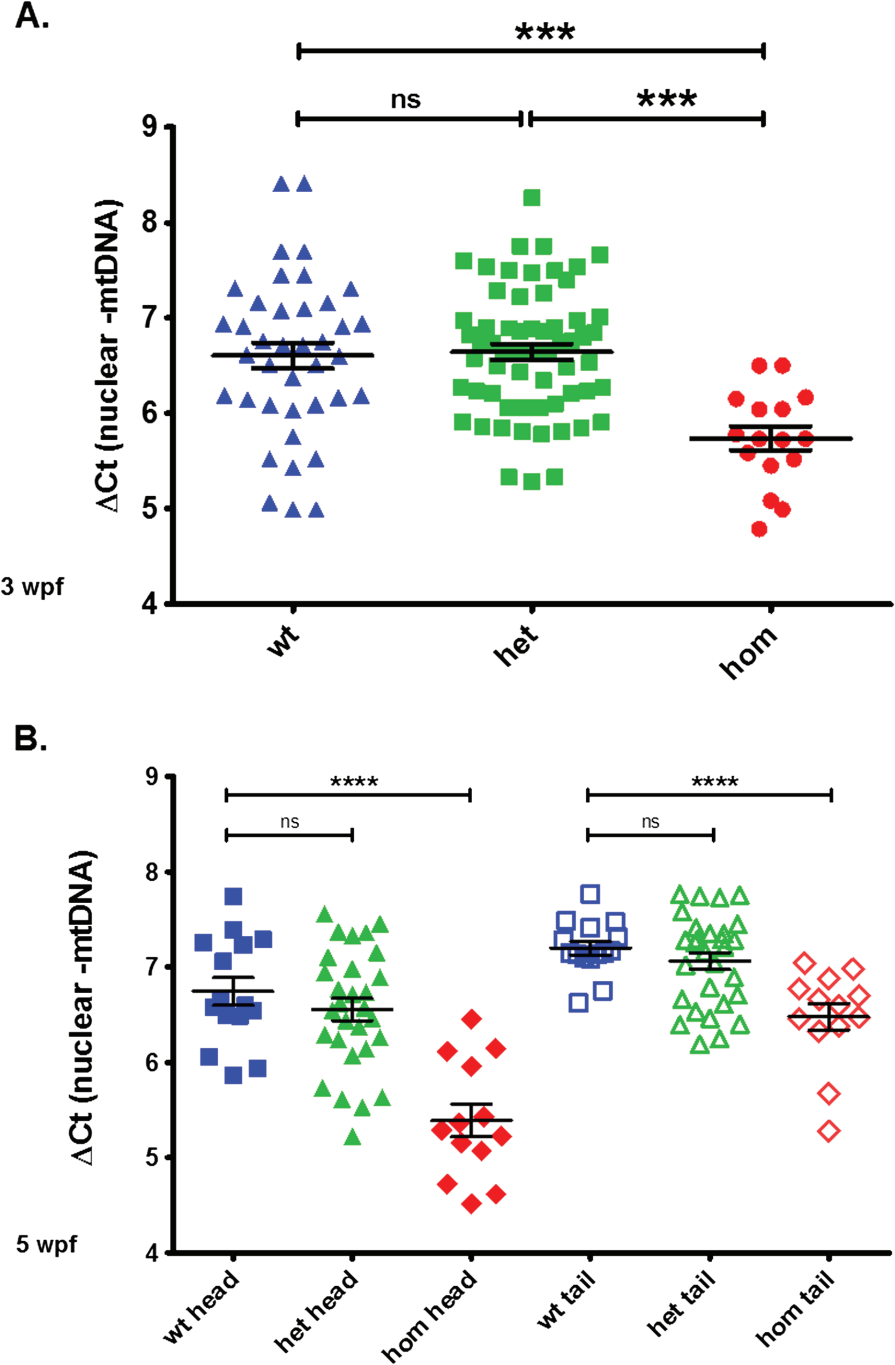
The mtDNA amount is significantly reduced in juvenile *dguok^−/−^* zebrafish. The mtDNA levels in heterozygous mutants were similar to the wt. The relative amounts of mtDNA were calculated as mean ΔCt values of the difference in cycle threshold (Ct) of the mitochondrial encoded gene ND1 minus the Ct of the nuclear gene *ef1α*. **(A)** mtDNA levels at 3 weeks of age (DNA isolated from entire fish). **(B)** mtDNA levels at 5 weeks of age in head/CNS and tail/skeletal muscle. The difference between wt and homozygous fish becomes more pronounced in the CNS with increased age.

As *DGUOK* mutations cause mtDNA depletion in humans, we measured relative mtDNA to nDNA levels in mutant and wt fish. At the age of 3 weeks we observed a significant 2-fold reduction of mtDNA levels in homozygous mutants. The 3-week-old fish appear normal despite having only 50% of the wt levels of mtDNA. The mtDNA amount in heterozygous animals did not differ from the wt (Fig. 2A, 117 fish tested in total).

At the age of 5 weeks we measured the mtDNA levels in the tail (skeletal muscle tissue) and in the head (brain tissue) of wt, heterozygous and homozygous mutant fish (Fig. 2B, 57 fish tested in total). The difference between wt and homozygous animals was increased compared with the difference detected at 3 wpf, especially in brain tissue. Heterozygous animals had similar mtDNA levels as wt animals. As some of the patients with *DGUOK* mutations were reported to have mtDNA deletions (11), we also performed long-range polymerase chain reaction (PCR) to amplify the mtDNA in mutant fish but detected no mtDNA deletions. In adult fish we detected a 4-fold higher mtDNA amount in wt compared with homozygous adults in tail muscle and brain (see Fig. 5, untreated fish). The mtDNA levels in adult mutants stayed constant up to the time of death. In fish that had to be culled because of the loss of the ability to swim no dip in the mtDNA copy number was observed before death.

### Nucleoside supplementation

**Figure 3:**
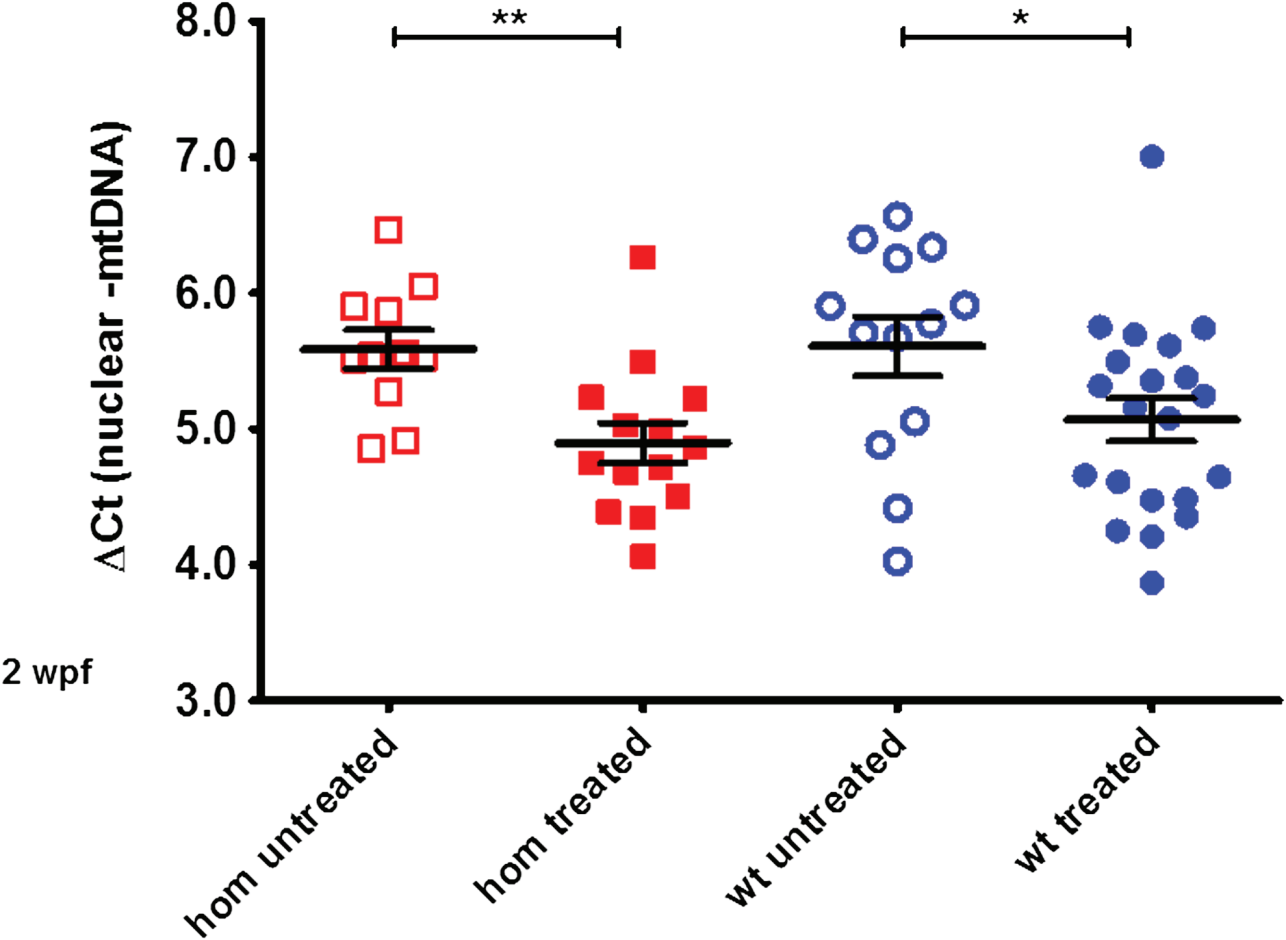
Supplementation with deoxyguanosine alone reduces mtDNA levels. The mtDNA amount is significantly reduced in wt and homozygous mutant juvenile fish by a 2 week treatment with 50 μm deoxyguanosine alone (total *N* = 25 homozygous, 34 wt animals, *P* < 0.05 for wt and *P* < 0.01 for homozygous fish, respectively).

Previously it has been shown in cultured cells that supplementation with 50 μm deoxyguanosine alone was sufficient to significantly increase the mtDNA copy number in fibroblasts from patients with *DGUOK* mutations (18). Therefore we used the same concentration for supplementation in our *dguok* mutant zebrafish. We treated offspring of a cross of *dguok* heterozygous parents (offspring should be a mixture of wt, heterozygous and homozygous animals) with 50 μm dGuo from 0 hpf onwards for 2 weeks. Initially we chose this time point, as by this age a difference between mtDNA levels in wt and mutant fish should be detectable (see Fig. 2). After 2 weeks of supplementation, the juvenile fish were culled, gDNA was isolated, the fish were genotyped and mtDNA levels were determined in wt and homozygous animals only (N: between 25 and 34 fish per group). Although the numbers of animals in each treatment group were not high, we observed that in the zebrafish animal model, in contrast to cultured patient fibroblasts, dGuo alone significantly reduced the copy number in mutant and wt animals and therefore the supplementation experiment with dGuo alone was stopped (Fig. 3).

**Figure 4:**
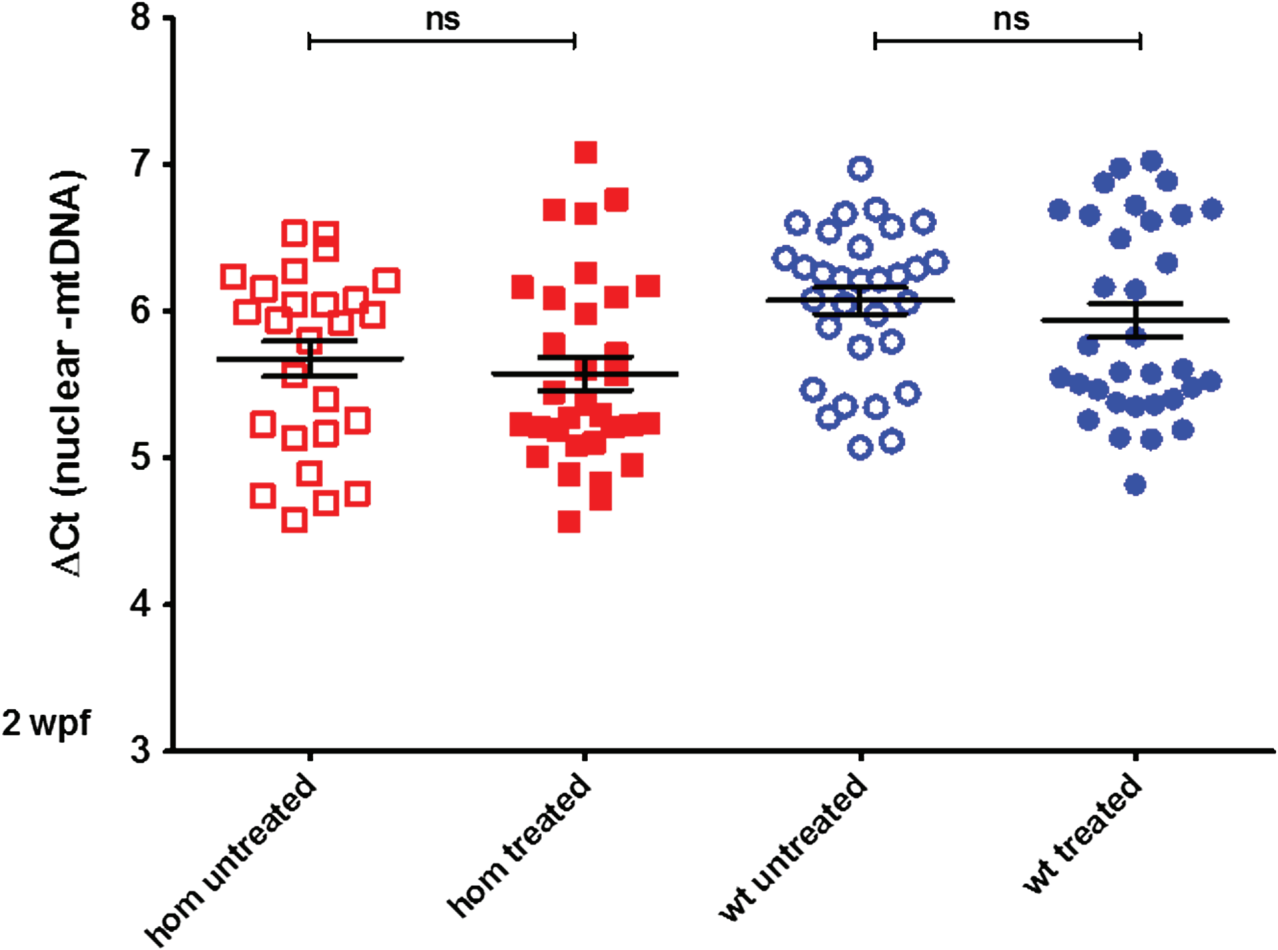
Sup plementation with deoxyguanosine and deoxyadenosine in juvenile fish. MtDNA levels do not change significantly in wt and homozygous mutant juvenile fish after a 2 week treatment with 50 μm deoxyguanosine and 50 μm deoxyadenosine (total number of untreated/treated animals: *N* = 26 and 32 homozygous, 31 and 33 wt animals). The differences between the homozygous untreated and treated group, as well as between the wt untreated and treated group, are not statistically significant.

Next, we decided to supplement the fish with a combination of both dAdo and dGuo for 2 weeks, starting again 0 hpf as previously. This time, the mtDNA levels did not decrease but we could not detect a significant increase either (Fig. 4). In comparison to the difference in mtDNA content seen earlier in Figure 3 after the single nucleoside treatment there was a lack of difference now in Figure 4 between treated and untreated fish. At this age, however, the mtDNA to nuclear DNA ratio difference between wt and homozygous mutants is significant but not very pronounced, and the variability between individual fish is relatively high (see Fig. 2). We suspected that this high variability among individual fish may obscure a small increase in mtDNA levels caused by the nucleoside supplementation.

**Figure 5:**
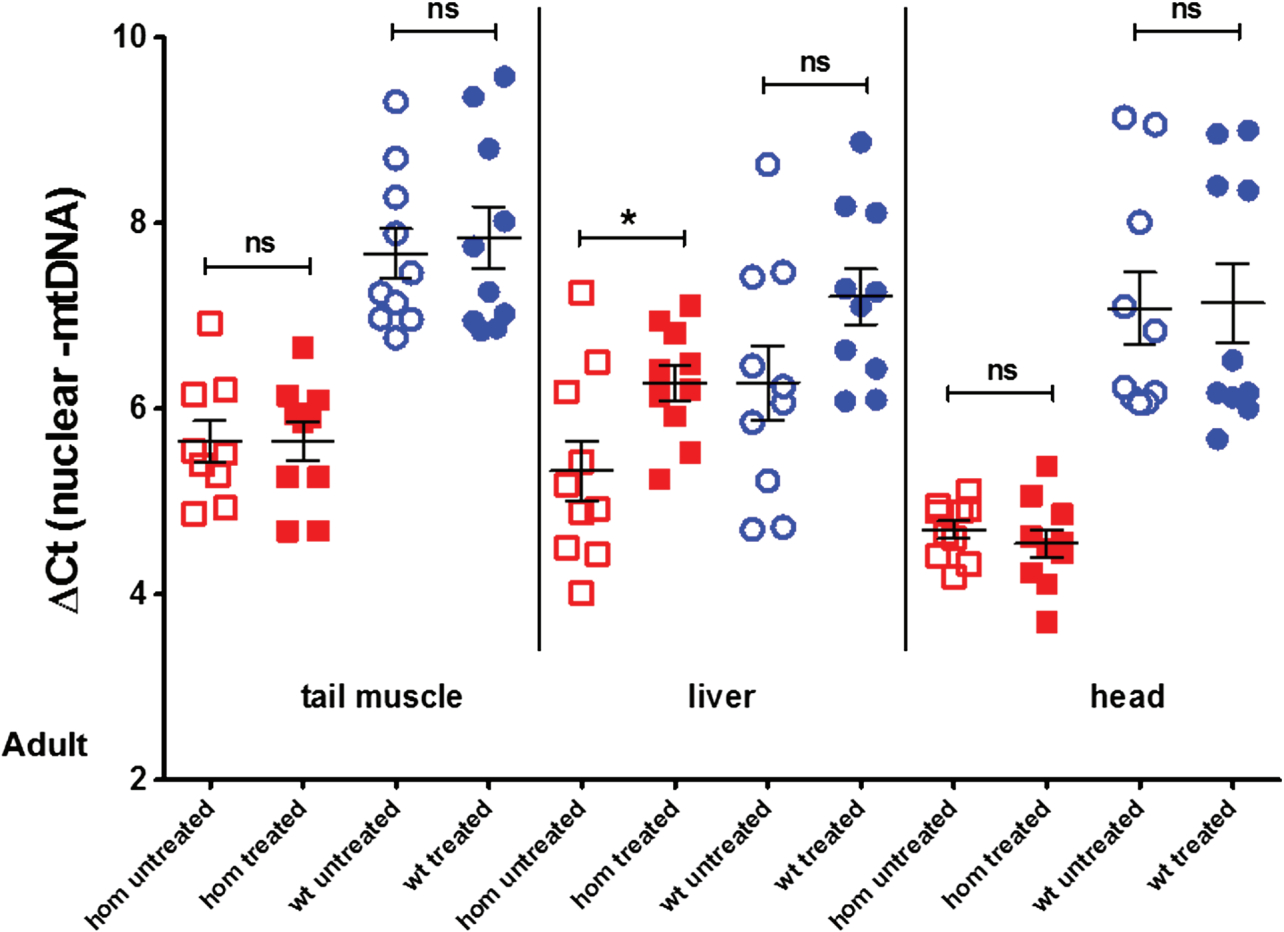
mtDNA levels in wt and homozygous adult fish after a 3 week treatment with 50 μm deoxyguanosine and 50 μm deoxyadenosine. Forty adult fish between the ages of 6 and 9 months were treated (10 fish per category); skeletal muscle, liver and heads were dissected after the treatment period for DNA extraction. The treatment induced a significant increase of mtDNA levels in the livers of mutant animals (*P*< 0.05); the mtDNA levels also increased in the livers of treated wt animals but the difference was not significant. Differences between untreated wt and untreated homozygous mutants were statistically significant in all three tissues (*P* < 0.0001, data not shown).

As we had noticed in preliminary experiments that the difference in mtDNA levels between *dguok* mutant and wt fish was more pronounced in adult fish, we next tried if supplementation with dGuo and dAdo in adult fish can lead to a restoration of the mtDNA levels. We treated adult wt and *dguok* homozygous fish with 50 μm dAdo and dGuo for 3 weeks. After the supplementation period we analysed mtDNA levels in the head (brain), tail (skeletal muscle) and the liver of treated and untreated fish. We detected a slight but significant increase in mtDNA levels in the liver of supplemented mutant fish but not in skeletal muscle or brain (Fig. 5). Difference in mtDNA levels between untreated wt and homozygous animals is most pronounced in the CNS (4-fold decrease, Fig. 5). In addition, we also analysed the protein level of mitochondrial RC complexes by western blot in treated and untreated adult fish (Supplementary Material, Fig. S4). We observed a slight increase in the amount of porin/VDAC1 and complex 4 and 5 but the increase was not significant. This might be due to the highly variable protein levels between individual fish. We could not detect any mtDNA deletions in either the treated or the untreated adult fish.

## Discussion

Animal models for MDS are essential for testing novel compounds and assessing whether they could be suitable for treating patients. Previous work from other laboratories suggested that good strategies for restoring mitochondrial dNTP pools are either bypassing the enzyme defect (17,19,23) or by increasing substrate availability (substrate enhancement 9,18). So far, the only *in vivo* work proving the efficacy of either dNMP or deoxynucleoside supplementation was performed in Tk2 H126N knock-in mice (9,19) and focused on the pyrimidine nucleotide half of the mitochondrial dNTP metabolism. To our knowledge, no mouse model for defects of the purine phosphorylating enzyme dGK has been published yet. Therefore, we decided to create a zebrafish model and investigate whether supplementation of the substrate nucleosides reverses the depletion of mtDNA levels in the mutants, as it was previously shown *in vitro*. We established a *dguok* mutant zebrafish line carrying a frameshift mutation in exon 3 leading to a premature translation termination. Although we were not able to verify protein expression, we expect that no fully functional dGK enzyme was expressed in the homozygous mutants. In contrast to humans with *DGUOK* mutations, the dGK-deficient zebrafish had lower mtDNA levels than their wt siblings but displayed no visible phenotype up to the age of ~10 months. mtDNA levels were at 50% of the wt levels in juvenile *dguok*^−/−^ fish, dropped further to ~25% in adulthood. MtDNA levels showed tissue-specific differences and reflecting the clinical presentation in patients, lower levels were detected in brain and liver tissue. However, this decrease of mtDNA amounts did not cause a visible phenotype in the homozygous mutants. One reason for this could be compensation by the cytoplasmic enzyme deoxycytidine kinase (dCK). dCK phosphorylates deoxyribonucleosides and nucleoside in the cytosol as part of the cytosolic salvage pathway. In cells that are dividing and replicating their nuclear DNA, nucleotides can be imported from cytosol into mitochondria (reviewed in 8). Zebrafish embryos and larvae grow fast and have rapid cell division, especially during the first days of development (24). This could ensure that enough dNTPs can be imported into mitochondria during this period. Another reason for the lack of phenotype in the *dguok* mutant fish during the early period of life could be the fact that there is no substantial increase in mtDNA copy number in the first 5 days of life in zebrafish embryos (25). Combined with the import of nucleotides from the cytosol it might explain why zebrafish compensate better for dGK deficiency than humans. All *dguok*^−/−^ fish eventually died or had to be culled for animal welfare reasons before the age of 1 year. The cause of the sudden deterioration of health or death remained unclear.

No zebrafish model has been described yet for the other enzyme of the mitochondrial salvage pathway, TK2. A zebrafish model for *POLG* mutations developed a much more severe phenotype than our *dguok^−/−^* fish with mtDNA depletion, smaller body size and death around the age of 4 weeks (26).

We attempted to trigger a phenotype in the *dguok*^−/−^ embryos by exposing offspring of an incross of *dguok*^+/−^ parents to S-(4-Nitrobenzyl)-6-thioinosine, an inhibitor of equilibrative nucleoside transporters (ENTs), up to the age of 5 dpf. Human ENT1 is localized on the cell surface as well as on the mitochondrial membrane and facilitates the uptake of nucleosides into mitochondria (27). We intended to block the uptake of nucleosides from the cytosol into mitochondria of the *dguok^−/−^* fish and thereby force the cells to rely more on their mitochondrial dNTP pools and thus on the enzymatic activity of dGK. However, no morphological phenotype was observed in the *dguok^−/−^* fish as a consequence of ENT inhibition. It may be possible though that S-(4-Nitrobenzyl)-6-thioinosine is not taken up from the water by zebrafish embryos or does not inhibit the zebrafish transporters at the concentrations used by us, as to our knowledge S-(4-Nitrobenzyl)-6-thioinosine has not been tested in zebrafish yet.

Although the *dguok* mutant fish did not display any visible morphological changes, the decreased mtDNA levels provided us with an experimental readout to assess the efficacy of chemical compounds as potential treatment for MDS. First, we attempted to increase the mtDNA levels in the *dguok* mutant fish by adding dGuo alone similar to the experiments performed in cell culture (18). Furthermore, studies in dGK-deficient cells showed that mainly dGTP levels were affected by *DGUOK* mutations (28) and were critical for the maintenance of mtDNA levels, suggesting that increasing the availability of dGuo could be enough to restore mtDNA copy number. However, we noticed that, in contrast to cultured fibroblasts, the mtDNA levels in zebrafish dropped during the supplementation with dGuo alone. It may be that increasing the concentration of just one of the substrate nucleosides introduces an imbalance into the dNTP pool and thereby interferes with mtDNA replication and as a consequence reduces the mtDNA copy number. This result prompted us to supplement the *dguok* mutant fish with both purine nucleosides instead. In this experiment we did not detect any drop of mtDNA levels in juvenile fish; however, we were not able to obtain a significant increase of mtDNA levels either. As the mtDNA levels varied considerably between individual animals, it might be likely that small changes are difficult to detect.

As the difference in mtDNA levels between wt and *dguok* deficient fish is much more pronounced in adult fish, we decided to supplement adult fish with both purine nucleosides. After 3 weeks of supplementation we detected an increase of mtDNA levels but only in the liver. MtDNA levels in tail skeletal muscle and brain did not change. Longer treatment of adult fish or life-long supplementation from 0 hpf onwards might increase mtDNA level also in other tissues in the *dguok^−/−^* fish or might be able to extend the life span of the mutants. The tissue-specific effect of the substrate enhancement therapy has previously been shown also in the mouse model of Tk2 deficiency (9). Similar to our results, deoxycytosine and deoxythmidine supplementation in the Tk2 mutant mouse led to a stronger increase of mtDNA levels in liver (and other internal organs that we did not study in zebrafish) compared with brain (9). In the Tk2 mutant mice, however, in contrast to the *dguok^−/−^* fish, mtDNA depletion was partially rescued in skeletal muscle. Purine nucleotides may be less stable than pyrimidines, and therefore higher doses may be required in dGK deficiency in order to obtain a recovery from mtDNA depletion. In addition, it has previously been shown that in mice and rats, mitochondrial turnover is faster in the liver compared with tissues such as brain and muscle, suggesting that this may be a factor in why we see the greatest change in mtDNA in the liver with a short treatment (29,30). The tissue-specific differences in the efficiency of the rescue of the mtDNA depletion need to be kept in mind if nucleoside supplementation will be tested in humans in the future. However, as the *DGUOK* patients mainly present with a hepatocerebral phenotype, a rescue of the mtDNA depletion in liver might still be beneficial.

In summary, zebrafish are a good *in vivo* model to study the effect of nucleoside supplementation in mtDNA maintenance diseases and may be further investigated. Other mtDNA maintenance defects might require supplementation with all four nucleosides in order to avoid dNTP pool imbalances. Based on our results, dosing nucleosides needs to be finely tuned and optimized, and results obtained *in vitro* in cell culture may not always be transferable to animal models.

## Materials and Methods

### Zebrafish husbandry

All zebrafish (*D. rerio*) used were of the *golden* background and were maintained according to standard protocol (24). All experiments were conducted in-line with the UK Home Office regulations with approval from the local Named Animal Care and Welfare Officers.

### sgRNA synthesis

The Crisprscan website (31) was used to identify target sites in exon 3 of the *dguok* gene in zebrafish (Genbank accession number for the sequence of zebrafish *dguok*: NM_001100091). sgRNA was produced through a cloning-free method by following a previously published protocol (32,33). The two selected guide RNA target sequences were gG18NGG-70AGGTGTAGGACCAGCGCTTCGGG and gG18NGG-86 CGCAGGAGTGTGTCCGGCAGCGG. The sgRNA was diluted to 300 ng/μl with 2 μm Cas9 protein (New England Biolabs Ltd, Hitchin, UK), 2 M KCl and 0.05% phenol red and heated to 37°C for 5 min. Embryos were injected at the one cell stage with 1 nl of the sgRNA/Cas9 protein mixture. Injected F0 fish were raised to adulthood and then outcrossed with golden fish in order to check for germline transmission of the mutation. Offspring of this outcross were raised as F1 generation and genotyped by fin clipping at the age of 3 months.

### Extraction of gDNA from zebrafish

gDNA was extracted and purified using the Qiagen Blood and Tissue kit (Qiagen, Hilden, Germany) with EconoSpin All-In-One Mini Spin Columns (Epoch Life Sciences Inc., Missouri City, TX, USA). DNA concentrations were measured with a Nanodrop 2000 spectrophotometer (Thermo Scientific).

### Zebrafish genotyping

*dguok* exon 3 was amplified from gDNA via PCR with primers designed to cover the region of interest (see primer table). PCR product was digested at 60°C with the restriction enzyme BstAPI (New England Biolabs) that cuts the wt sequence into two fragments and leaves the homozygous sequence uncut.

### Measurement of mtDNA levels in zebrafish

MtDNA levels were determined using qPCR following the protocol previously published by Rahn *et al*. (26) using primers for a mitochondrial gene, NADH dehydrogenase-1(*ND1)* and a nuclear gene, elongation factor 1 alpha (see primer table). The relative amount of mtDNA was calculated at the Ct value difference of the two genes. All samples were run in triplicate. Statistical analysis (Analysis of variance, *t*-test) was performed in the GraphPad Prism software.

### Long-range PCR in zebrafish

Long-range PCR assays were performed in order to determine the presence of deletions in the zebrafish mtDNA. Primers zf mtDNA 6395 fwd and zf mtDNA 15929 rev were used to amplify a fragment of the zebrafish mtDNA corresponding to the fragment containing the most common mtDNA deletions detected in patients. PrimeSTAR GXL polymerase (Takara Bio Europe, Saint-Germain-en-Laye, France) and 100 μg template DNA were used for the long-range PCR; the primer sequences are listed in the primer table.

### RNA isolation, Reverse transcription polymerase chain reaction (RT-PCR) and gene expression analysis in zebrafish

Trizol® (ThermoFisher, Waltham, MA, USA) was used to isolate RNA from adult zebrafish tail tissue following the manufacturer’s instructions. Residual gDNA was removed by incubating the RNA with DNAseI (DNA-free™ DNA Removal Kit, Ambion, Waltham, MA, USA). The High-Capacity cDNA Reverse Transcription Kit (Applied Biosystems, Waltham, MA, USA) was used to synthesize cDNA from the isolated RNA following the manufacturer’s instructions; 2 μg total RNA was used for reverse transcription. RT-PCR was performed using the primers listed in the table. quantitative reverse transcription polymerase chain reaction (qRT-PCR) (Biorad Iight-cycler equipped with a MyIQ detection system) was performed in triplicates (technical replicates with the same cDNA) using the iTaq™ Universal SYBR® Green Supermix (Bio-Rad Laboratories Ltd, Hercules, CA, USA) and the primer pairs listed in the primer table. All primer pairs except the *dguok* primers have been published previously (25,26). Beta-actin was used as housekeeping gene to normalize gene expression.

### Nucleoside supplementation

Two different nucleoside supplementation treatments were performed. The first consisted of 50 μm 2^ʹ^-Deoxyguanosine monohydrate (D7145, Sigma-Aldrich, St. Louis, MO, USA**)** dissolved in system water. The second consisted of both 50 μm 2′-Deoxyguanosine monohydrate (D7145, Sigma-Aldrich, St. Louis, MO, USA**)** and 50 μm 2′-Deoxyadenosine monohydrate (D7400, Sigma-Aldrich, St. Louis, MO, USA) with 5 μm of EHNA hydrochloride (E114, Sigma-Aldrich, St. Louis, MO, USA) dissolved in system water. EHNA hydrochloride was added to inhibit the breakdown of deoxyadenosine by the enzyme adenosine deaminase.

### Nucleoside supplementation in juvenile zebrafish

Heterozygous (+/−) *dguok* mutants (c.351_352delGCinsCCTG) were group spawned and embryos collected in petri dishes. Embryos were either supplemented with nucleoside solution or system water (control). The water was changed daily, reapplying the treatment, and any dead embryos were removed. At 5 dpf embryos were placed into 1 litre plastic spawning tanks and water changed every 48 h and fish fed three times daily. At 2 wpf the embryos were culled and collected in accordance with the home office guidelines via a schedule 1 method.

### Nucleoside supplementation in adult fish

Adult homozygous (−/−) *dguok* mutants (c.351_352delGCinsCCTG) were placed in 2 litre tanks, with treatment solution (*n* = 5) or with system water (control) (*n* = 5). Wt (control) fish were placed in two tanks, one with treatment (*n* = 5) and one with system water (*n* = 5). Adults were only supplemented with both 50 μm deoxyguanosine and deoxyadenosine (and EHNA). The water was changed every 48 h, treatment reapplied and fish fed three times daily, twice with dry flake (Tetra, Blacksburg, VA, USA) and once with brine shrimp (*Artemia*). After 3 weeks, the fish were collected and culled in accordance with the UK Home Office guidelines via a schedule 1 method. Immediately after the fish had been culled they were dissected and tail muscle, liver and heads were collected and frozen before analysis. The health of the fish was monitored throughout the treatment. Supplementation of adult fish was performed twice with a total of 10 animals per group.

### Immunoblotting

Muscle tissue from the tail of adult zebrafish were homogenized in RIPA buffer with protease inhibitors. A total protein of 30 μg were loaded on 4–12% SDS–polyacrylamide gels (NuPAGE™ 4–12% Bis-Tris Protein Gels, ThermoFisher, Waltham, MA, USA), transferred to a PVDF membrane with an iBlot®2 PVDF Mini transfer stack (ThermoFisher, Waltham, MA, USA) and subsequently probed with antibodies recognizing NDUFA9 (Abcam plc, Cambridge, UK ab14713, 1:500), MTCO1 (Abcam plc, Cambridge, UK ab14705, 1:500), porin/VDAC1 (Abcam plc, Cambridge, UK ab15895, 1:1000), ATP5A (Abcam plc, Cambridge, UK ab14748, 1:1000), GAPDH (Anti-GAPDH (14C10) rabbit mAb #2118 Cell Signalling Technology, Danvers, MA, USA) or β-actin (Sigma-Aldrich, St. Louis, MO, USA A1978, 1:2000). Quantification of protein levels was done using the Fiji (ImageJ) software.

### ENT1 inhibition

Heterozygous (+/−) *dguok* mutants (c.351_352delGCinsCCTG) were spawned and embryos collected. Embryos (*n* = 100) were exposed to a range of concentrations (1, 5, 10 and 50 μm) of S-(4-Nitrobenzyl)-6-thioinosine (Sigma-Aldrich, St. Louis, MO, USA, N2255) or DMSO (D8418, Sigma-Aldrich, St. Louis, MO, USA) (vehicle control) at 1 dpf. The larvae were monitored for the first 5 dpf for any phenotypic abnormalities and treatment solution was reapplied every 48 hpf. At 5 dpf a sample of embryos (*n* = 16) was collected from each treatment group and genotyped as described above. We subsequently repeated the treatment once with treatment concentrations of 10 μm, 50 μm S-(4-Nitrobenzyl)-6-thioinosine or DMSO starting with embryos at 0 dpf.

## Supplementary Material

Supplementary Data
