# Supplementary material for: Nucleoside supplementation modulates mitochondrial DNA copy number in the dguok −/− zebrafish
Source: Hum Mol Genet. 2018 Nov 14;28(5):796–803. doi: 10.1093/hmg/ddy389 (PMC6381312; doi:10.1093/hmg/ddy389)
Supplement: Supplementary Data [file ddy389_supp.zip › Munro_supplementary file.docx]

**Supplementary figure 1**

**
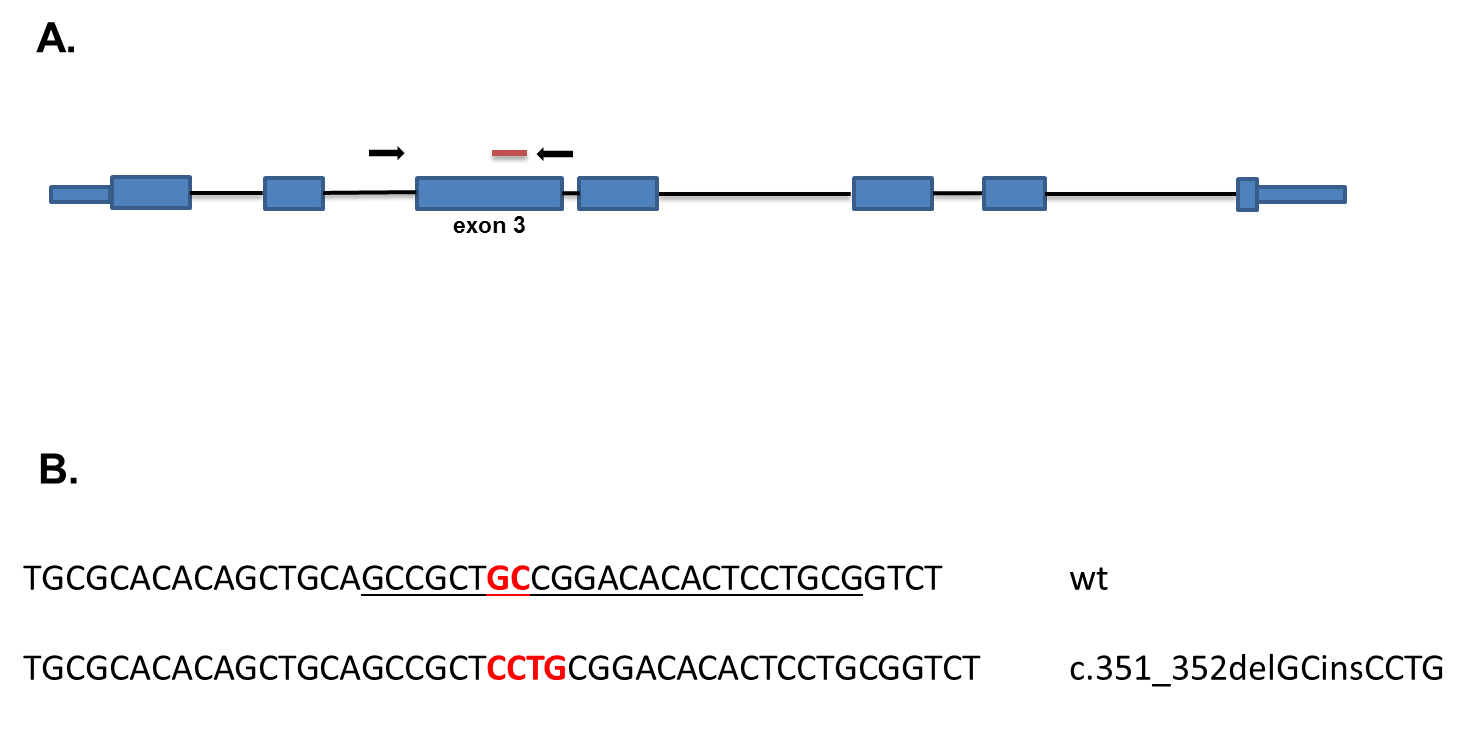
**

**C.**

*Xenopus laevis* --------------MKSQTTKPLQQSISTNLSSNKEMQVKRLSVEGNIAVGKSTFLRLLS 46

*Danio rerio* -----MIIKTAK---RLLSSSALFC-S-PRNLMSTNNYIKRVSIEGNIAVGKSTFARLLQ 50

*Homo sapiens* MAAGRLFLSRLRAPFSSMAKSPLEGVS-SSRGLHAGRGPRRLSIEGNIAVGKSTFVKLLT 59

*Mus musculus* MAAGRFLLRRLRASFRSPLRNALVDAP-HARAMHDGGGPRRLCIEGNIAVGKSTFVKLLM 59

*Rattus norvegicus* MAAGRFLLRRLRASFRSQPRNALVDAP-RARGMHDGGGPRRLCIEGNIAVGKSTFVKLLT 59

*Xenopus laevis* NTFQEWSFATEPLKKWQNIQSTSFQTTTSSKPPMDNLLQLMYDDPKRWSYTFQTFSCMSR 106

*Danio rerio* NACPDWDVIAEPVSKWQNVDQTPPTAS-SPQPCTSNLLEMMYRDPKRWSYTFQSFSCMSR 109

*Homo sapiens* KTYPEWHVATEPVATWQNIQAAGTQKA-CTAQSLGNLLDMMYREPARWSYTFQTFSFLSR 118

*Mus musculus* KTHPEWQVATEPIAEWQNIQAAGAQKD-GTSKRLGNLLEMMYQEPARWSYTFQTLSFMSR 118

*Rattus norvegicus* KTHPEWQVATEPIATWQNVQAAGTQKD-STSRRLGNLLDMMYQEPARWSYTFQTLSFMSR 118

**↓**

*Xenopus laevis* FKIQIQPLSEPVLKQQ-EHVQIFERSVYSDRYIFAKTLYELQHLNEMEWTLYQEWHTFLI 165

*Danio rerio* LRTQLQPLPDTLLRSRGGAVRVYERSVYSDRYIFALNMFALGCINSTEWAVYQDWHSFLI 169

*Homo sapiens* LKVQLEPFPEKLLQAR-KPVQIFERSVYSDRYIFAKNLFENGSLSDIEWHIYQDWHSFLL 177

*Mus musculus* LKVQLEPIPGRLLQAE-KSVRVFERSVYSDRYIFAKNLFENGSLSDIEWHIYQDWHSFLL 177

*Rattus norvegicus* LKVQLEPTPGRLLQAD-TSVRVFERSVYSDRYIFAKNLFENGSLSDVEWHIYQDWHSFLL 177

*Xenopus laevis* QEFSRRVALDGIIYLWATPEKCFERLQRRARKEEKTLQLQYLEKLHDQHESWLTKKTTEV 225

*Danio rerio* EQFGHRVQLEGIIYLRASPQMCLERLNRRARVEEQEIELDYLEKLHTRHEDWLINKTTTL 229

*Homo sapiens* WEFASRITLHGFIYLQASPQVCLKRLYQRAREEEKGIELAYLEQLHGQHEAWLIHKTTKL 237

*Mus musculus* QEFANRLLLHGFIYLQASPQVCMERLYQRDREEEKGIELAYLQQLHSQHEDWFINKTTKL 237

*Rattus norvegicus* QEFEDRLLLHGFIYLQASPQVCMERLCQRGREEEKGIELAYLKQLHGQHEDWFINKTTKL 237

*Xenopus laevis* SFENMKNIPVLLLNVEEDFENNSAAGDELNNRVKAFVAGL- 265

*Danio rerio* HFEQLMKVPVLVLDAEVAFEQNPEVQDCLLSKVRDFLSQL- 269

*Homo sapiens* HFEALMNIPVLVLDVNDDFSEEVTKQEDLMREVNTFVKNL- 277

*Mus musculus* HFEALQHVPVLVLDVTEDFSENAARQEELMGQVNTFMRNL- 277

*Rattus norvegicus* HFEALRHVPVLVLNISEDFSENAAKQEELMGQQGDSDNRRE 278

**
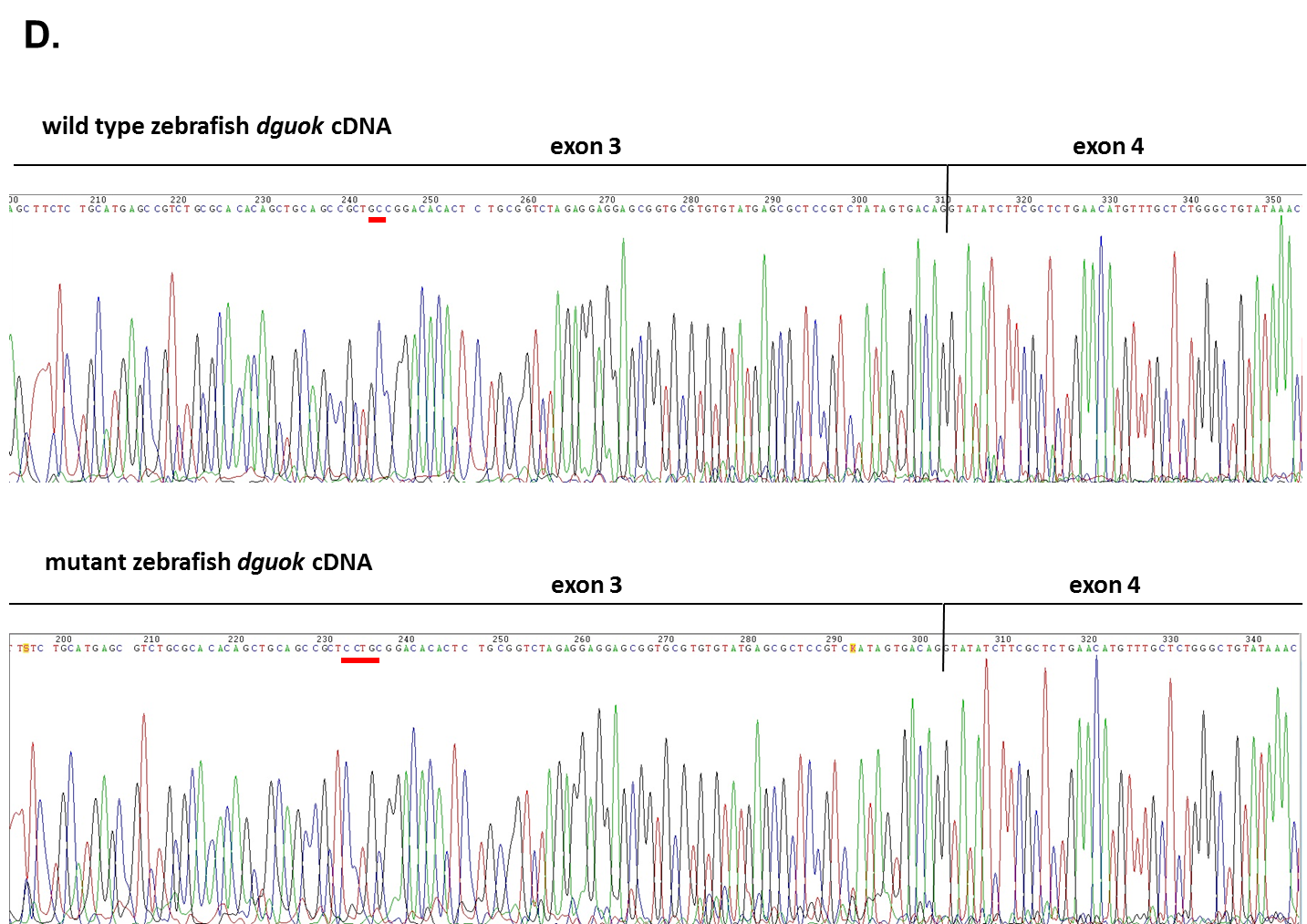
**

**
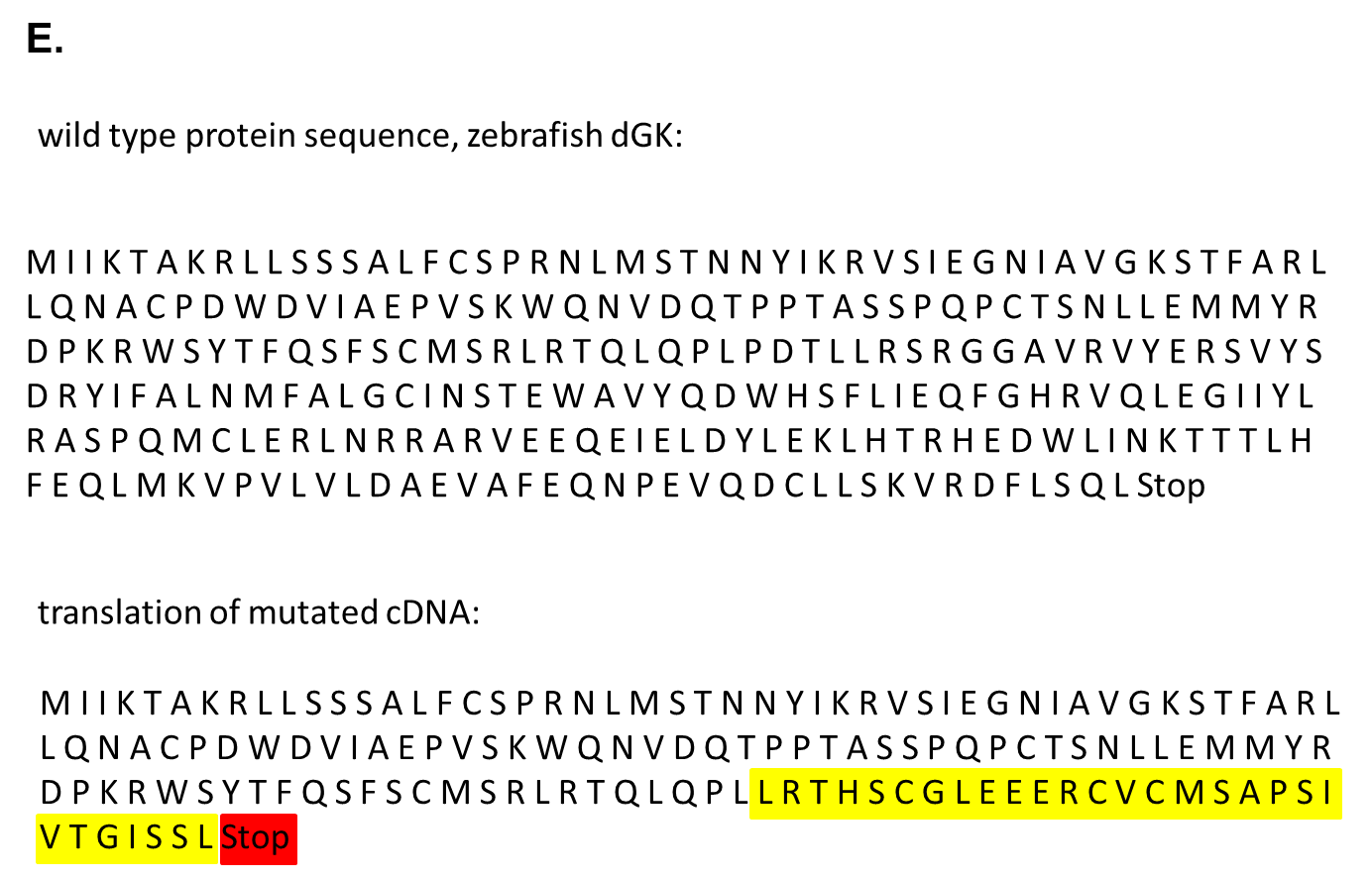
**

**Supplementary figure 2**

**
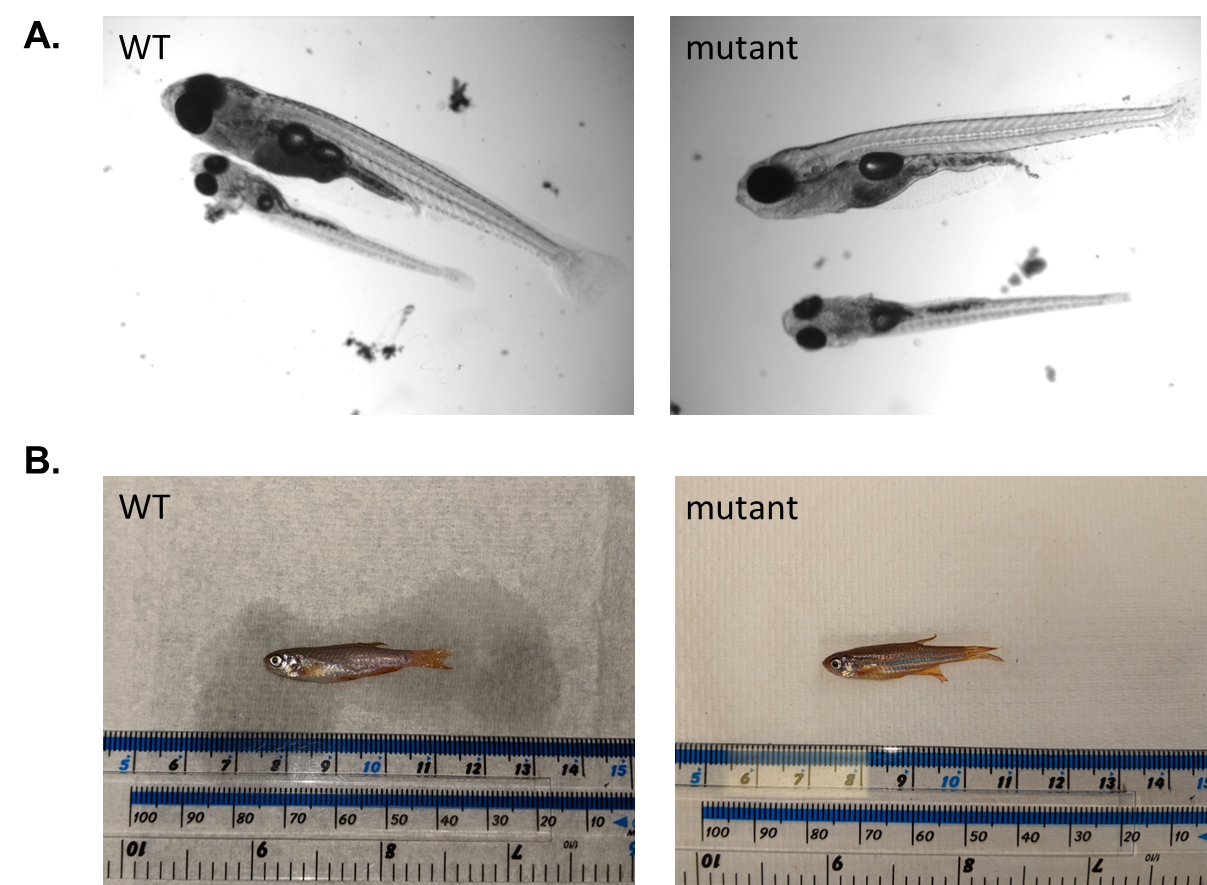
**

**Supplementary figure 3**

**
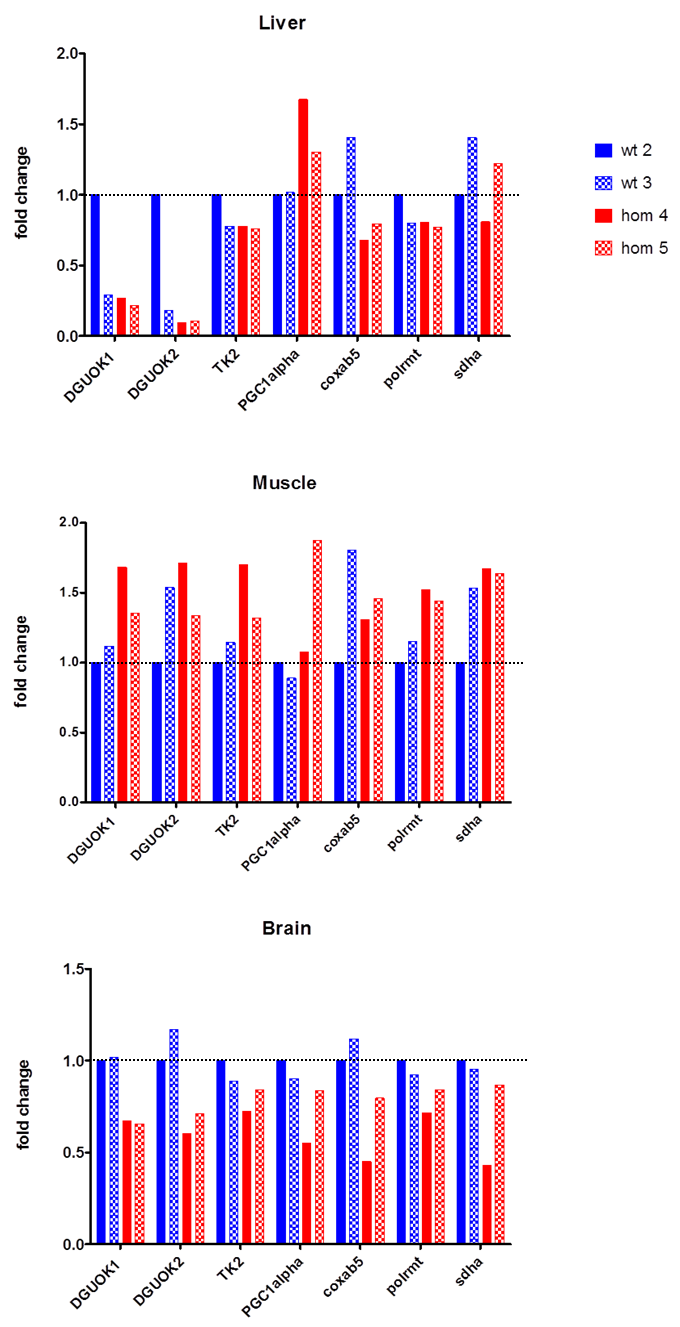
**

**Supplementary figure 4**

**
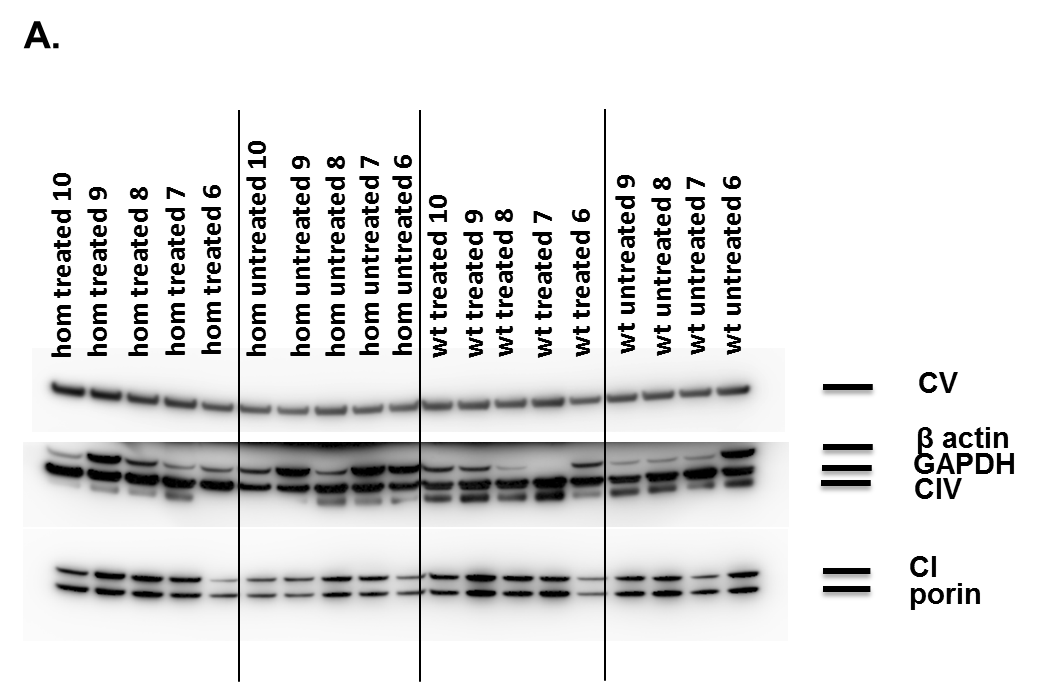
**

**
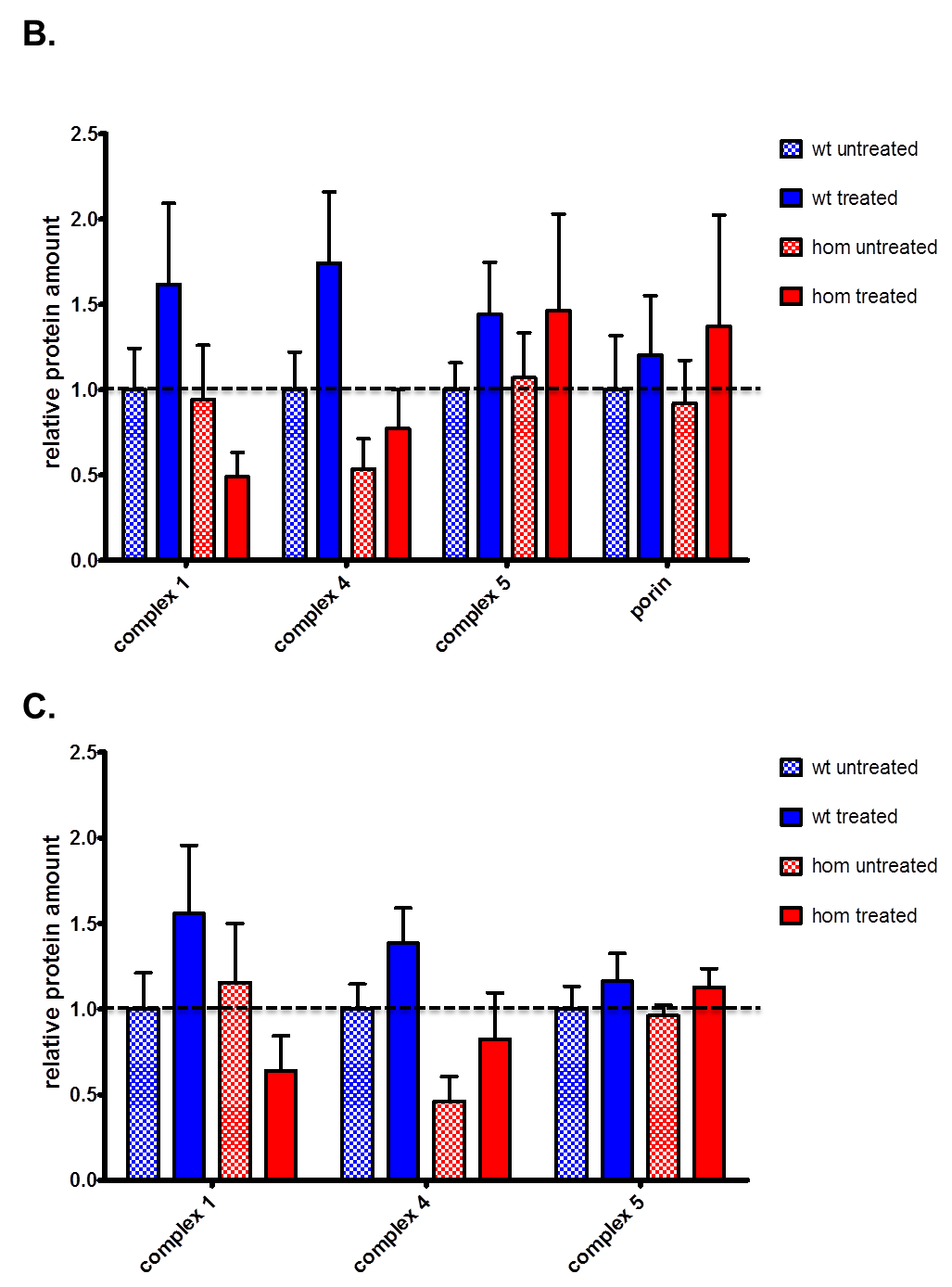
**

**Supplementary table:**

Primer sequences

| **Primer name** | **Sequence** |
| --- | --- |
| beta actin f | 5'-TGACAGGATGCAGAAGGAGA |
| beta actin r | 5'-GCCTCCGATCCAGACAGAGT |
| 18S RNA f | 5'-AGCGTGCGGGAAACCACGAG |
| 18S RNA r | 5'-AAGCCGCAGGCTCCACTCCT |
| Thymidine kinase 2 f | 5'-CCTGTATGAGGACTGGCTGA |
| Thymidine kinase 2 r | 5'-TCTGTTCTCCTCAAACTGATGC |
| POLG-1f | 5'-GGTGACCAGTGAAGACCGATA |
| POLG-1r | 5'-GTCCACTGCGCTAAAGAAGG |
| TFAM f | 5'-GCGAAAGATTGCCCAGCAGT |
| TFAM r | 5'-TTGTCGTTTTTCCTCCGCAAA |
| ND1 cDNA f | 5'-AGCCTACGCCGTACCAGTATT |
| ND1 cDNA r | 5'-GTTTCACGCCATCAGCTACTG |
| ndufs4 f | 5'-TGTAGGCTGGCAGAGGGACA |
| ndufs4 r | 5'-GACAGGCCGAAACAGGATGG |
| sdha f | 5'-TGGTATGCCGTTCAGCCGTA |
| sdha r | 5'-GGCCAAGTCTTTGGCATTGG |
| uqcrc2 f | 5'-GACCTCACGGGAAGGGTGAA |
| uqcrc2 r | 5'-TCAGTGTGCTGGTGCTGCTG |
| cox5ab f | 5'-GGTCACCGGAGCTTCAGGAT |
| cox5ab r | 5'-TCGAGCCGAGAGGTAGAAAAACC |
| atp5a1 f | 5'-TTCTTGGAGCCGACACTGGA |
| atp5a1 r | 5'-CGAACACCACAACACCAACG |
| polrmt f | 5'-ACCCGCTGCCGCCTTATTTT |
| polrmt r | 5'-TCCAGCGAGCTCTGCTTCTTC |
| pgc1a f | 5'-GGCCCAGCGAGCCAAACCAA |
| pgc1a r | 5'-TGGCTTTGTGAGGAGGCGTGG |
| zDGUOK qPCRex1f | 5'-atattgcggttgggaagtcc |
| zDGUOK qPCRex2r | 5'-gtttggtccacattctgcca |
| zDGUOK qPCRex6f | 5'-cgtgttggttctggatgctg |
| zDGUOK ex7rev | 5’-cttcagcagcagaaacatcaga |
| zDGUOK intr2f | 5’- TAACCAACCTCTCTCTTCGTGT |
| zDGUOKex3rev | 5’- CACTATAGACGGAGCGCTCATA |
| ND1 fwd (mtDNA copy number) | 5’- GGGCACCCATACCCATGCCCTAT |
| ND1 rev (mtDNA copy number) | 5’- TGCGCTACAGCTCGTAAGGC |
| Elf1α fwd (mtDNA copy number) | 5’- AAGCCGCTGAGGTAAGCGTTCAAC |
| Elf1α rev (mtDNA copy number) | 5’- TTGAGCCGAGAAACGCGTGCTG |
| zf mtDNA 6395 fwd | 5’- taaacactcggccaccctac |
| zf mtDNA 15929 rev | 5'- tttatccatgttggggtttagg |

**Supplementary figures:**

**Figure 1:** **The *dguok* gene in zebrafish.** A. Schematic view of the zebrafish *dguok* gene. The guide RNA target site used for creating the mutant line is located in exon 3 (red). Arrows: PCR primers used for genotyping. B. *dguok* mutation in our zebrafish mutant line. The guide RNA target site is underlined, the deleted and inserted nucleotides are in red. C. alignment of protein sequences. The zebrafish protein is 55% identical and 72% similar to its human counterpart. Amino acids highlighted in green are conserved among all species aligned here. The arrow marks the position of the mutation. D. Screenshots of the *dguok* cDNA sequences of wild type and mutant zebrafish. The location of the mutation is underlined in red. E. Wild type zebrafish dGK protein sequence and truncated sequence obtained by translating the mutated cDNA sequence. The new amino acids introduced by the mutation are highlighted in yellow.

**Figure 2:** **Images of *dguok* mutant and wild type fish**. A. Zebrafish at the age of 3 weeks post fertilisation. Left: offspring of a cross of two wild type fish. Right: offspring of a cross of two *dguok*^+/-^ fish. B. Adult wild type (left) and homozygous *dguok* mutant fish (right).

**Figure 3:** **Gene expression analysis** in two adult wild type and two adult homozygous mutant fish in liver, tail muscle and brain tissue. Gene expression was normalised to beta actin and to the first wild type fish. DGUOK1 is a PCR product spanning exons 1 and 2 (5’ of the mutation) and DGUOK2 is a PCR product spanning exons 6 and 7 (3’ of the mutation). Beta-actin was used as housekeeping gene; 18S RNA was not included as the values varied considerably among individual fish in brain tissue.

**Figure 4:** **Protein analysis in adult fish.** A. Western blot on skeletal muscle tissue of adult wild type and *dguok^−/−^* zebrafish (untreated or treated with 50µM deoxyguanosine and 50µM deoxyadenosine). The following antibodies used to detect OXPHOS complexes: complex V – ATP5A, complex IV – MTCO1, complex I – NDUFA9. GAPDH has been used as total protein loading control, porin (VDAC) as control for the amount of mitochondria present in the tissue. Non-muscle beta actin has been used to detect the amount of non-skeletal muscle tissue present in the tails of the dissected fish.

B. Quantification of protein levels in relation to GAPDH as loading control. The values plotted here represent the average of 7 fish (6 in the untreated wt group). The average level detected in the wild type untreated fish has been set to 1. The protein level differences observed between treated and untreated homozygous fish are not significant, as there is a high variability between the protein levels observed in individual fish from each group. The same is true for the difference between wild type untreated and homozygous untreated animals. Error bars represent SEM.

C. Quantification of protein levels in relation to porin (VDAC) as control for the amount of mitochondria present in the tissue. The values plotted here represent the average of 7 fish (6 in the untreated wt group). The average level detected in the wild type untreated fish has been set to 1. Error bars represent SEM.
